# The anterior temporal lobes are critically involved in acquiring new conceptual knowledge: Evidence for impaired feature integration in semantic dementia^☆^

**DOI:** 10.1016/j.cortex.2013.10.006

**Published:** 2014-01

**Authors:** Paul Hoffman, Gemma A.L. Evans, Matthew A. Lambon Ralph

**Affiliations:** Neuroscience and Aphasia Research Unit (NARU), University of Manchester, UK

**Keywords:** Conceptual knowledge, Semantic memory, Learning, Frontotemporal dementia, Anterior temporal lobe

## Abstract

Recent evidence from multiple neuroscience techniques indicates that regions within the anterior temporal lobes (ATLs) are a critical node in the neural network for representing conceptual knowledge, yet their function remains elusive. The hub-and-spoke model holds that ATL regions act as a transmodal conceptual hub, distilling the various sensory-motor features of objects and words into integrated, coherent conceptual representations. Single-cell recordings in monkeys suggest that the ATLs are critically involved in visual associative learning; however, investigations of this region in humans have focused on existing knowledge rather than learning. We studied acquisition of new concepts in semantic dementia patients, who have cortical damage centred on the ventrolateral aspects of the ATLs. Patients learned to assign abstract visual stimuli to two categories. The categories conformed to a family resemblance structure in which no individual stimulus features were fully diagnostic; thus the task required participants to form representations that integrate multiple features into a single concept. Patients were unable to do this, instead responding only on the basis of individual features. The study reveals that integrating disparate sources of information into novel coherent concepts is a critical computational function of the ATLs. This explains the central role of this region in conceptual representation and the catastrophic breakdown of concepts in semantic dementia.

## 1 Introduction

Conceptual knowledge for objects comprises a diverse set of information about their sensory qualities, motor plans and verbal associations. How are these disparate sources of information linked to form a concept? According to one influential view, originally proposed by Wernicke (Wernicke, 1900; as cited in Eggert, 1977), conceptual knowledge for objects arises from the co-activation of their sensory-motor properties within a network of modality-specific processing regions that are widely distributed throughout the cortex (Barsalou, 2008, Martin, 2007, Pulvermuller, 2001). This approach makes two key predictions concerning the breakdown of conceptual knowledge under brain damage. First, damage to a single, modality-specific region should give rise to knowledge deficits that disproportionately affect properties in that modality and, by extension, categories of objects for which the affected modality is particularly central (Capitani et al., 2003, Mahon and Caramazza, 2009, Warrington and Shallice, 1984). So, for example, damage to regions of inferior parietal cortex involved in representing skilled actions should impair knowledge of how objects are manipulated and lead to a disproportionate deficit for tools (Buxbaum & Saffran, 2002). The second prediction concerns global, pan-modal conceptual impairments. According to Wernicke and his modern counterparts, these should only occur as a result of global cortical damage, because only damage to all of the modality-specific regions would be sufficient to produce a global impairment. This prediction is challenged by the neurodegenerative syndrome of semantic dementia (SD). SD patients suffer from a global conceptual knowledge deficit that affects all categories of object and word (Hoffman and Lambon Ralph, 2011, Lambon Ralph et al., 2007) and all sensory-motor modalities (Bozeat et al., 2000, Bozeat et al., 2002, Luzzi et al., 2007, Piwnica-Worms et al., 2010), yet the cerebral atrophy and hypometabolism that gives rise to this debilitating impairment is not global: it is focused bilaterally on the anterior ventrolateral and polar portions of the temporal lobes (Galton et al., 2001, Mion et al., 2010). Evidence from functional neuroimaging (Binney et al., 2010, Visser and Lambon Ralph, 2011) and transcranial magnetic stimulation (Pobric et al., 2007, Pobric et al., 2010) in neurologically-intact participants confirms that ventrolateral anterior temporal lobe (ATL) areas are involved in all forms of conceptual processing irrespective of the modality of the information or the category of object probed. The crucial role of this area in transmodal semantic representation also fits with recent *in vivo* tractography data demonstrating the convergence of multiple white-matter pathways into the ATL. Such results indicate that this region's structural connectivity is ideal for blending different sources of verbal and nonverbal information into integrated, coherent concepts (Binney, Parker, & Lambon Ralph, 2012).

To account for the global, pan-modal involvement of the ventrolateral ATLs in conceptual knowledge, we have developed an alternative framework for conceptual knowledge termed the “hub-and-spoke” model (Lambon Ralph et al., 2010, Patterson et al., 2007, Pobric et al., 2010, Rogers et al., 2004). This model holds that in addition to modality-specific sources of information (“spokes”) and their inter-connections, representation of conceptual knowledge requires an integrative “hub”. The hub uses information from the modality-specific spoke regions to develop modality-invariant, conceptual representations that capture deeper patterns of conceptual similarity across all sensory-motor and verbal modalities. These integrated representations are necessary because similarity in any particular sensory-motor domain is, at best, only a partial guide to conceptual similarity (Dilkina and Lambon Ralph, 2013, Lambon Ralph et al., 2010, Smith and Medin, 1981). For example, though apples and bananas have different shapes, colours and tactile properties and are manipulated in different ways, the conceptual system must be able to recognise that they are similar types of object. In addition, true conceptual representation requires the integration of properties that are experienced in different times and situations, and representation of the complex, non-linear relationships between the concept's verbal and nonverbal modality-specific properties and its conceptual significance (see Lambon Ralph et al., 2010 for more detailed discussion of these issues). The hub-and-spoke framework holds that the ATL hub provides this critical aspect of conceptual representation through the formation of representations that integrate information from all sensory-motor-verbal domains. When this region is damaged, as in SD, the result is a breakdown in the complex boundaries that define different concepts, such that semantic decisions come to be made on the basis of superficial characteristics rather than their deeper conceptual properties. For example, SD patients may reject “emu” as an example of a bird but simultaneously over-extend the concept to accept “butterfly” (Lambon Ralph et al., 2010, Mayberry et al., 2011).

Previous work on the function of the ventrolateral ATLs has focused on their role in representing existing knowledge and its progressive deterioration as a result of ATL atrophy in SD (e.g., Binney et al., 2010, Rogers et al., 2004). The hub-and-spoke framework also predicts that the ATLs play a key role in the acquisition of novel concepts (Rogers & McClelland, 2004). There is already some support for this idea from electrophysiological studies in primates. The response properties of anterior inferior temporal neurons change as monkeys learn novel associations between visual stimuli, suggesting a role for this region in the acquisition of concepts (Albright, 2012). In the present study, we tested this hypothesis in humans by studying acquisition of new conceptual knowledge in patients with SD. The hub-and-spoke model predicts that the ATLs are critical for integrating the various sensory features of an object into a unified, coherent conceptual representation that can be generalised to new exemplars. We tested this prediction by training SD patients to recognise novel visual stimuli as members of two categories. Previous research has shown that SD patients are able to apply well-defined rules to classify novel stimuli, when the classification rule is provided by the experimenter (Koenig, Smith, & Grossman, 2006). Here, we tested the patients' ability to acquire more complex category structures that could not be captured by a simple rule and when no information about the nature of the categories was supplied by the experimenter.

**Fig. 1:**
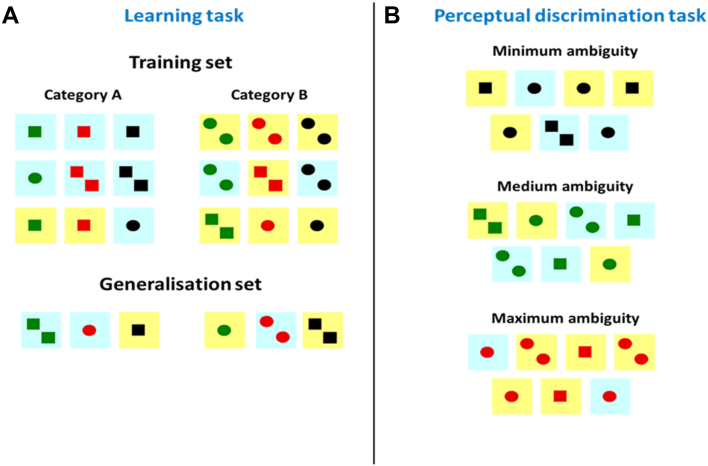
Experimental Stimuli. (A) Stimuli were divided into two categories according to a family resemblance structure. The top row of the training set comprises exemplars that possessed all three typical features of their respective category. The remaining exemplars possessed two typical features of the category and one feature associated with the opposing category. Stimuli in the generalisation set were not presented during training but retained for subsequent test. (B) Perceptual discrimination test. Each trial comprised three identical pairs of stimuli and a lone one odd-one-out. The three levels of ambiguity manipulated the number of features the odd-one-out shared with the pairs.

The structure of the two categories (shown in Fig. 1A) was designed such that optimal performance could only be achieved by acquiring integrated representations of the various typical characteristics of each category. When presented *en masse* as in Fig. 1, it is easy to discern the features associated with each category. Members of Category A usually contained squares while those in B contained circles, though there were exceptions in both categories. The same was true for the number of shapes (members of A usually contain one shape) and the colour of the background square (usually blue for A). The colour of the internal shapes, though perceptually salient, was not diagnostic of category. This category structure, in which a number of features are associated with each category but no single feature is diagnostic, is termed a family resemblance structure and is characteristic of object categories in the real world (Rosch and Mervis, 1975, Smith and Medin, 1981, Wittgenstein, 1953). Within such a structure, it is impossible to classify with complete accuracy by learning only about a single feature dimension. Optimum performance instead requires participants to form integrated representations that include second-order statistical information about the feature conjunctions that characterise each category, allowing them, for example, to correctly class an exemplar with two circles as a member of Category B, even if it has a blue background. We predicted that forming such integrated representations is a key function of the ATLs and, therefore, that SD patients would be impaired in learning the categories.

We deliberately selected an abstract, novel set of stimuli with little perceptual similarity to objects in the real word, to ensure that pre-existing conceptual knowledge would not influence the learning process. However, the novel stimuli's underlying family resemblance structure meant that they shared several important attributes with real conceptual categories.

1. Items in a category shared a number of typical characteristics but no single feature was diagnostic of category membership (e.g., most creatures that fly are birds but there are also a number of flightless birds and some non-bird creatures that can fly).
2. While there were no individual diagnostic features, the conjunction of a number of typical features was a good guide to category membership (e.g., a creature that lays eggs and has feathers and a beak is likely to be a bird, even if it cannot fly).
3. Some features, though salient, were not useful in determining category membership (e.g., the colour of a creature is not helpful in deciding whether it is a bird or not).

Our hypothesis was that the computational challenges posed by these complex, natural categories are met by the ATLs, which form integrated conceptual representations that allow us to categorise items based on the overall summation of their characteristics rather than relying on a single defining feature. We predicted that SD patients would be impaired in their ability to acquire these integrated representations, leading to an over-reliance on individual features to guide their category decisions.

## 2 Method

### 2.1 Patients and background testing

**Fig. 2:**
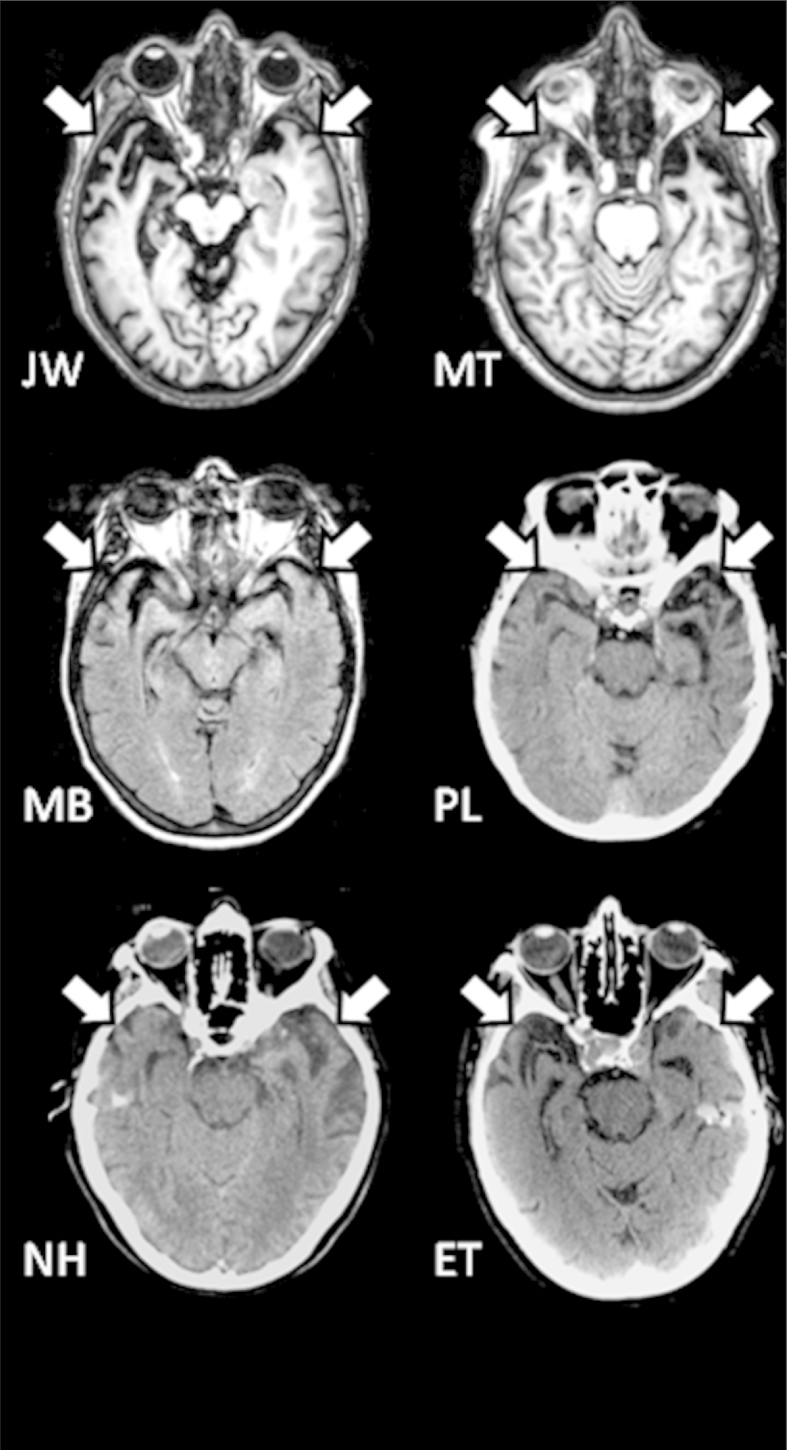
Structural imaging. Structural MR or CT images for patients, indicating anterior temporal atrophy in each case. No images were available for P.W., though an MR scan report confirmed that this patient also had anterior temporal damage.

Seven patients with SD were recruited from memory clinics in northwest and southwest England. All met published diagnostic criteria for SD (Gorno-Tempini et al., 2011, Hodges et al., 1992), in that they presented with pan-modal conceptual knowledge deficit that affected receptive and expressive tasks. Other aspects of cognition were preserved in all but the most severe cases: patients were well-oriented in time and space and presented with fluent, grammatically correct speech. However, the case-series was intended to span the full range of severity in semantic performance and one of the most severe cases (N.H.), while initially presenting with a selective semantic impairment, had begun to show signs of decline on other cognitive tasks at the time of the study. Structural neuroimaging indicated bilateral atrophy of the anterior temporal region in each case (see Fig. 2).

Patients completed a battery of standard neuropsychological tests. Conceptual knowledge was assessed using elements of the Cambridge Semantic Battery (Bozeat et al., 2000), consisting of tests of picture naming, spoken word–picture matching, pictorial semantic association (the Camel and Cactus Test) and verbal fluency for six semantic categories. All seven patients performed below the normal range on all tests. As expected, there was a broad range of impairment in conceptual knowledge from mild to very severe (see Table 1; patients are ordered from mild to severe based on word–picture matching scores). General dementia severity was assessed with the Addenbrooke's Cognitive Examination-Revised (Mioshi, Dawson, Mitchell, Arnold, & Hodges, 2006) and the Mini Mental State Examination (Folstein, Folstein, & McHugh, 1975). Visuospatial processing was tested using the Rey figure copy and two subtests from the Visual Object and Space Perception battery (Warrington & James, 1991). Patients also completed tests of digit span (Wechsler, 1987) and Raven's coloured progressive matrices (Raven, 1962). These tests revealed the expected pattern of relative preservation of other cognitive functions in most cases. The case-series included two severely impaired patients: N.H. and E.T. At time of testing, N.H. had begun to show signs of more general cognitive decline. In contrast, E.T. performed strikingly well on the non-semantic tasks, despite severe semantic impairment. We included both patients in the case-series in order to assess the effects of severe conceptual knowledge impairment on learning; however, it is possible that concomitant deficits may have affected N.H.'s performance. Importantly, the other six patients all demonstrated preservation of the basic perceptual and cognitive functions necessary to complete the category learning task. Raven's progressive matrices were particularly informative in this regard. Like the experimental task described below, it involves abstract coloured geometric shapes. It also has a strong problem-solving element and requires understanding the notion of similarity relationships between stimuli. All of the patients except N.H. performed well on this test.

**Table 1:** Demographic information and background neuropsychology.

Test	Max	J.W.	M.T.	M.B.	P.L.	P.W.	N.H.	E.T.	Control mean (range)
Sex		F	F	F	F	M	F	F	
Age		63	61	61	73	73	69	80	
School-leaving age		16	16	15	15	17	16	14	

*Cambridge Semantic Battery*									
Picture naming	64	43	44	32	22	8	11	0	62.3 (57–64)
Word–picture matching (chance level = 6/64)	64	61	50	48	43	33	19	14	63.8 (63–64)
Semantic association (CCT)	64	49	37	30	30	34	24	NT	59.1 (51–62)
Category fluency (six categories)	–	53	50	37	26	22	14	8	95.7 (61–134)

*General neuropsychology*									
ACE-R	100	62	67	67	56	41	29	43	93.7 (85–100)
MMSE	30	29	27	27	23	23	17	21	

*Visuospatial*									
Rey figure copy	36	34	36	35	31	34	27.5	29.5	34.0 (31–36)
VOSP number location	10	8	10	10	7	10	10	7	9.4 (7–10)
VOSP cube analysis	10	10	10	10	9	10	8	10	9.7 (6–10)

*Attention/Executive*									
Digit span forward	–	6	7	6	8	5	4	7	6.8 (4–8)
Digit span backward	–	6	6	4	5	4	4	6	4.8 (3–7)
Raven's coloured progressive matrices	36	32	34	31	31	34	13	29	

ACE-R = Addenbrookes Cognitive Examination – Revised (Mioshi et al., 2006); MMSE = Mini-mental state examination (Folstein et al., 1975); VOSP = Visual Object and Space Perception battery (Warrington & James, 1991); CCT = Camel and Cactus test (Bozeat et al., 2000).

### 2.2 Experimental stimuli

Twenty-four abstract visual stimuli were created based on those used by Waldron and Ashby (2001). Stimuli varied on four dimensions: background colour, internal shape, number of shapes and shape colour. Background colour, shape and number were all relevant for categorisation. These dimensions each had two possible values (e.g., shape: circle or square) and we refer to these as “features”. The shape colour dimension had three possible values (red, black and green) and was irrelevant for classification. A family resemblance structure was used to divide the stimuli into two categories, arbitrarily labelled A and B (see Fig. 1A). Each of the three relevant dimensions had a feature reliably associated with each category, though no single dimension was fully diagnostic of category. Eighteen exemplars were presented during the category learning task. Three exemplars in each category possessed all of the three features associated with the category (i.e., the typical background, typical number and typical shape for their category, shown in the top row of Fig. 1A). The remaining exemplars had two features that were typical of their category, while the remaining feature was more strongly associated with the opposing category. Six exemplars were not presented at all during the learning task but were retained to later test the participants' ability to generalise their learning to novel exemplars.

### 2.3 Category learning task

Patients completed a learning task over two sessions on consecutive days. Each learning session consisted of 144 trials. At the beginning of the task, patients were told that they would see some abstract patterns and would attempt to learn which ones were “A”s and which were “B”s. They were told that there was no simple rule for deciding but that it was something they would learn over time. On each trial, they saw one of the 18 exemplars, presented in the centre of a laptop computer screen on a white background. The letters A and B were presented in bottom left and right corners of the screen and the patient was asked whether the exemplar was an A or a B. They were then presented with a green tick if they decided correctly or a red cross if they chose the wrong category. Verbal feedback was also given at first so that patients understood the significance of the ticks and crosses. At no point were participants told which aspects of the stimuli to attend to or how to make their decisions. The 144 trials were divided into eight blocks, with each exemplar presented once in each block. For the second session, the patients were told that they were continuing the task they started the previous day and that the identity of the A's and B's had not changed.

To determine the degree to which participants were able to form integrated category representations, categorisation success during the second half of the second session was analysed in detail (72 trials). By this point, participants had completed 216 trials of the learning task, allowing them to form stable representations of the characteristics of each category.

### 2.4 Generalisation test

The generalisation test probed participants' ability to apply their acquired knowledge of the categories to a new set of stimuli comprised the same features but in novel combinations. This allowed us to rule out an alternative basis for task performance: namely, that participants had used an episodic memory strategy and attempted to memorise the correct category for each individual stimulus, rather than learning the underlying properties that characterised the two categories. We reasoned that knowledge of the underlying category structure would generalise to a new set of stimuli that participants had not seen during learning. In contrast, if participants had only learned the categories for the specific stimuli presented during learning, they would not be able to classify new stimuli at an above-chance level.

To test for generalisation, immediately after the second session participants were presented with six new exemplars, not presented during training. They were asked to classify them as before, though no feedback was given. Each of the six new exemplars was presented a total of four times.

### 2.5 Visual discrimination test

In a recent study, Barense, Rogers, Bussey, Saksida, and Graham (2010) demonstrated that SD patients can have difficulty discriminating between visual objects when they have many overlapping features. Specifically, patients were impaired when required to discriminate stimuli based on conjunctions of features, even in a purely perceptual task with no learning requirement. This raises the possibility that apparent deficits in learning could arise because SD patients have difficulty perceiving the stimuli correctly. To ensure that our patients were able to discriminate between the stimuli in our experiment, we tested them with a demanding odd-one-out task described by Barense et al. (2010).

On each trial, patients were presented simultaneously with seven exemplars from the learning study. The seven stimuli consisted of three identical pairs and one “odd-one-out” and patients were asked to point to the odd-one-out. There were three conditions of increasing difficulty. In the minimum ambiguity condition, the odd-one-out could be detected on the basis of a single stimulus dimension (e.g., in Fig. 1B, it is the only exemplar containing two shapes). In the medium ambiguity condition, it was necessary to perceive the conjunction of two dimensions to distinguish the odd-one-out (e.g., in Fig. 1B, only the odd-one-out has squares on a yellow background). Finally, in the maximum ambiguity condition, the odd-one-out could only be detected by integrating all three dimensions. The three conditions were intermixed and there were 105 trials in total. Patients completed the discrimination test at least two weeks after completing the learning task.

### 2.6 Control participants

Twelve healthy volunteers completed the learning and generalisation tests. They had a mean age of 69 years and educational level of 16.7 years, neither of which differed from the patients [*t*(17) < 1.9, *p* > .05]. Six different individuals completed the visual discrimination test. Their mean age was 68 and education was 16.0 years [not significantly different from patients: *t*(11) < 1.0, *p* > .05].

## 3 Results

Mean categorisation accuracy in the control group was 67% (standard deviation = 9.7%), which indicates that learning the family resemblance category structure under experimental conditions was challenging even for healthy participants, as expected from previous studies (Medin, Wattenmaker, & Hampson, 1987). SD patients also averaged 67% (standard deviation = 4.7%) and their accuracy was not significantly different to that of controls [*t*(17) = .15, *p* = .88]. Importantly, binomial tests indicated that all seven patients were significantly above chance in their categorisation performance (*p* < .0019). This indicates that all of the patients understood the nature of the task (i.e., they were not guessing) and were able to acquire some information about the novel stimuli. To determine the nature of the representations formed by our participants, we analysed performance on the final 72 trials of the learning task in more detail. These analyses revealed that learning in the SD group took a very different form to that seen in the control group, as we describe next.

### 3.1 Learning across stimulus dimensions

**Fig. 3:**
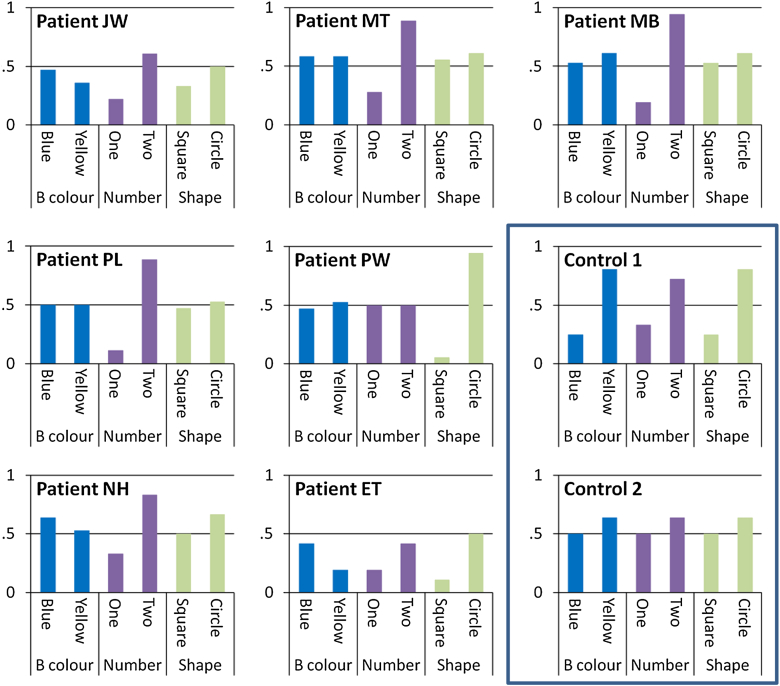
Individual performance profiles. Pattern of responding for all patients and two representative controls. The *y*-axis shows the probability of responding “B” to stimuli with each feature. All three dimensions were relevant for classification. Control 1 displayed an optimum pattern of learning, successfully learning the category–feature associations in each dimension. Control 2 was less successful but still displayed modest learning in all three dimensions. In contrast, patients were more likely to learn the category structure in only a single dimension. B colour = background colour.

Our key prediction was that SD patients would have difficulty forming integrated representations that included information about all three dimensions needed for optimal classification. To test this, we investigated how participants classified stimuli with each type of feature. Fig. 3 shows the data from each patient and, for comparison purposes, from two representative controls. Each participant's responses have been split according to the exemplar's features on each of the three critical dimensions. The *y*-axis shows how often the participant responded B to stimuli with each feature, so values close to one indicate items that were usually classed as B's and values close to zero show items that were usually classed as A's. Control 1 showed an optimal pattern of responding: she successfully acquired knowledge about the typical features in all three dimensions (this can be seen clearly by comparing her pattern of responses with the set of category members in Fig. 1A; for example, she correctly classified most of the circle exemplars as B's and the squares as A's). This control participant performed at over 90% accuracy during the final phase of learning. Control 2 achieved much poorer learning overall (60% accuracy) but showed a similar qualitative pattern. She also learned about all three dimensions equally, albeit to a much lesser extent. The pattern in the patients was rather different and indicates that they were unable to form coherent representations that combined all three dimensions. Four patients (M.T., M.B., P.L. and P.W.) learned about only one of the three critical dimensions, as indicated by strong differentiation and one dimension and a lack of discrimination on the other two dimensions. For example, P.W. classified all stimuli based on their shape, ignoring their number and background colour.^1^ The remaining three patients showed a more ambiguous pattern of performance, with weak learning on two stimulus dimensions.

To investigate these profiles in more detail, we calculated *d*′ scores for each participant. *D*′ is a signal detection measure that reflects a participant's tendency to give a particular response when presented with a particular type of stimulus weighed against their propensity to make the same response to other stimuli. We computed *d*′ scores that expressed a participant's sensitivity to the feature–category associations in each of the three dimensions. According to our predictions, SD patients should show strong learning (i.e., high *d*′ values) in one dimension but much weaker learning across the remaining dimensions. Controls were expected to display a more even pattern of learning across the three dimensions. Once *d*′ scores had been computed, an additional step was necessary to compare the results in the two groups. Since different participants learned about different aspects of the stimuli (e.g., compare patient M.T. with P.W.), a simple averaging of the *d*′ scores in each dimension would mask the true effects. Instead, we labelled the dimensions for each participant according to their *d*′ scores, with the dimension in which the greatest learning had occurred labelled as their strongest dimension (so M.T.'s strongest dimension was number, her second dimension was shape and her weakest dimension was background colour). We were then able to average *d*′ scores within each group based on the strongest, second and weakest dimensions of each individual.

**Fig. 4:**
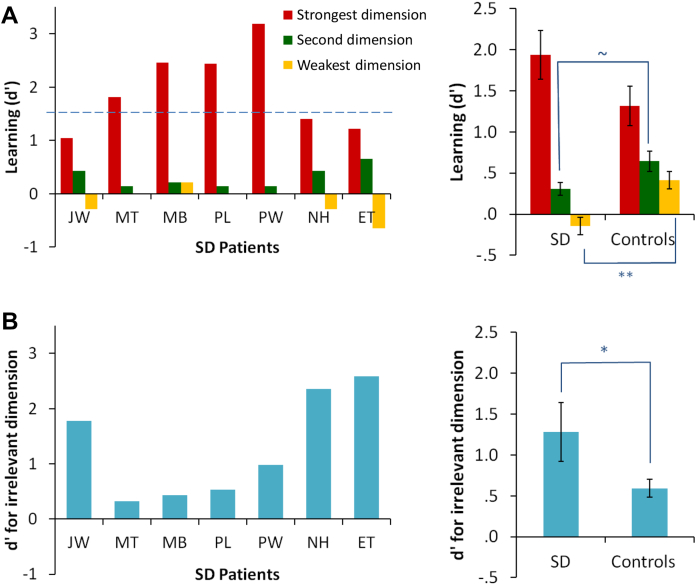
Sensitivity to stimulus dimensions. (A) Strength of learning (*d*′) on each dimension for each patient and the two groups (see text for details). The blue line indicates the optimum *d*′ for all three dimensions. Patients are arranged in descending order of semantic performance (word–picture matching). (B) *D*′ measure for the shape colour dimension, which was irrelevant for classification. The optimum *d*′ in this case is zero. * indicates *p* = .04; ** indicates *p* = .003 and ∼ indicates *p* = .07.

*D*′ scores are shown for each patient in Fig. 4A. It is important to note that interpretation of the *d*′ scores presented here is slightly different to most circumstances. In most studies, a particular stimulus feature is *always* associated with a particular response and optimum performance is signified by the maximum possible *d*′ value (typically between 3 and 4). Because of the family resemblance structure employed here, each feature was only associated with its typical category on 78% of trials. As a consequence, the optimum *d*′ score was lower: a participant classifying with 100% accuracy would have *d*′ scores of 1.52 for each dimension (indicated by the blue line in Fig. 4A). Scores higher than this indicate an over-extension of the learning in the strongest dimension, such that the information in this dimension was driving classification even for exemplars where the other two dimensions pointed towards a different category. This over-generalisation was present in four of the seven patients and is similar to the over-generalisation exhibited by SD patients when attempting to use their impaired conceptual knowledge of real objects (see Discussion). No patients demonstrated much learning in their second or weakest dimensions, in line with the prediction that they would be unable to form category representations that integrated all of the information required for optimum categorisation.

The mean *d*′ scores in each group can be seen in Fig. 4A. As expected, there was a large disparity between the strongest dimension and the remaining two dimensions in SD, with a more balanced pattern of learning across the three dimensions in the control group. A 3 (dimension) × 2 (group) ANOVA was performed on these data. There was a main effect of dimension [*F*(2,34) = 43, *p* < .001]. There was no effect of group but there was a highly significant interaction between dimension and group [*F*(2,34) = 6.83, *p* = .003]. Post-hoc *t*-tests indicated that SD patients showed significantly less learning on their weakest dimension than controls [*t*(17) = 3.44, *p* = .003]. There was also a trend towards poorer learning on the second dimension in SD patients, relative to controls [*t*(17) = 1.95, *p* = .07].

While the general pattern in the patient group was towards strong, single-dimension learning, we did observe some variation across patients, with J.W., N.H. and E.T. displaying a less clear pattern than the other four patients. To investigate these differences, we tested whether these patients' responses were influenced by the shape colour dimension, which was irrelevant for classification. We calculated a *d*′ measure of “learning” in this dimension in a similar manner to the other dimensions. Since this dimension was irrelevant to classification, the optimum *d*′ was 0. The results are shown in Fig. 4B. The four patients who achieved the most successful learning on their strongest dimension showed low *d*′ values, indicating that they were not influenced by the irrelevant dimension. However, patients N.H. and E.T., and to a lesser extent J.W., had higher *d*′ scores, indicating that their responses were incorrectly influenced by this dimension. This suggests a more severe impairment in these individuals, since their responses were guided by stimulus features that were not reliably associated with either category. In line with this hypothesis, the two patients with the most severe semantic deficit showed the largest effects (N.H. and E.T.). *D*′ scores in the SD group as a whole were also compared with those of the control group (see Fig. 4B). As a group, SD patients were more likely to be influenced by the irrelevant dimension than controls [*t*(17) = 2.26, *p* = .04].

### 3.2 Accuracy on critical “inconsistent” trials

The general picture emerging from the *d*′ analyses was that SD patients displayed relatively successful learning on their strongest dimension but were less successful in learning the category associations in the other two dimensions. This suggests that they failed to integrate the various stimulus features into a coherent conceptual representation. As a strong test of this interpretation, we re-analysed categorisation accuracy but now specifically considered trials on which an over-reliance on learning in one dimension would cause participants to choose the wrong category. Trials from the final period of learning were divided into two conditions for each participant:

1. Consistent trials: On most trials (78%), the feature on the strongest dimension indicated the correct category for the exemplar. On these trials, participants could categorise correctly even if they had only acquired knowledge in a single dimension.
2. Inconsistent trials: Due to the family resemblance structure, there were a minority of trials in which the feature in the participant's strongest dimension did not indicate the correct category. Participants could only give the correct response on these trials if they had also acquired some knowledge of the other two dimensions, which would direct them towards the correct response. Consequently, we expected SD patients to have particular difficulty on these trials, because it was not possible for them to select the correct category unless they had achieved integrated learning across multiple dimensions.

**Fig. 5:**
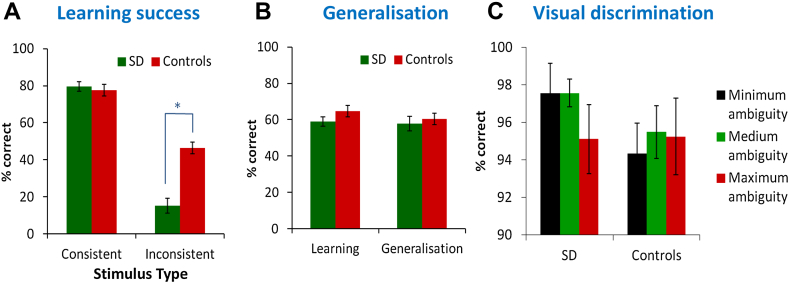
Response accuracy on each test. (A) Accuracy during the final phase of learning, with trials divided according to their consistency with each participant's most strongly learned dimension. (B) Accuracy in classifying generalisation stimuli introduced after the training period, compared with equivalent stimuli in the learning period. (C) Accuracy in perceptual discrimination at varying levels of complexity.

Fig. 5A shows correct responses in each condition, averaged within the two groups. The data were analysed with 2 × 2 mixed ANOVA that included condition and group. This revealed main effects of both group [*F*(1,17) = 10.7, *p* = .005] and condition [*F*(1,17) = 89, *p* < .001]. The condition effect indicates that both groups found the inconsistent trials more difficult. Critically, there was also a highly significant interaction [*F*(1,17) = 10.8, *p* = .004]. Post-hoc tests indicated that patients performed as accurately as controls on consistent trials (*t* < 1) but were substantially impaired on inconsistent trials [*t*(19) = 4.15, *p* = .001]. This supports the hypothesis that patients were less able to form representations that included information from multiple dimensions and instead responded solely on the basis of their strongest dimension.

### 3.3 Generalisation test

The generalisation test probed participants' ability to apply their acquired knowledge of the categories to novel stimuli. Performance on the new stimuli was above chance in both groups [one-tailed one-sample *t*-tests: SD patients: *t*(6) = 1.94, *p* = .05; Controls: *t*(11) = 3.19, *p* = .009]. We also compared performance on the generalisation stimuli with performance in the final block of the learning task, to assess how successfully learning transferred to new exemplars. For the purposes of this comparison, we excluded the six highly prototypical stimuli from the training set (i.e., the stimuli on the top row of Fig. 1A that possessed all three typical features for the category). These stimuli were considerably easier to classify because they possessed all three typical features. We excluded them because there were no equivalent stimuli in the generalisation set: all of the generalisation had at least one feature associated with the opposing category.

Performance for generalisation trials and equivalent learning trials is shown in Fig. 5B. A 2 × 2 ANOVA revealed no difference between learning and generalisation [*F*(1,17) = 1.79, *p* = .2], no effect of group [*F*(1,17) = .91, *p* = .4] and no interaction [*F*(1,17) = .59, *p* = .5]. Based on these findings, it is unlikely that either patients or controls were memorising the correct category for individual stimuli. Instead, they attempted to form more general representations of the characteristics of each category, which allowed them to generalise to new exemplars.

### 3.4 Visual discrimination test

The visual discrimination test measured participants' ability to perceive the conjunctions of features present in the stimuli and to discriminate between them. Patients and controls performed close to ceiling, even for the most demanding trials (see Fig. 5C). A 3 (condition) × 2 (group) mixed ANOVA comparing patients with controls revealed no main effect of either group [*F*(1,11) = 1.65, *p* = .2] or condition [*F*(2,22) = .38, *p* = .5] and no interaction [*F*(2,22) = .60, *p* = .6]. The performance of each individual patient was compared with the control group using the modified *t*-test (Crawford & Howell, 1998). No patient showed a significant impairment in any of the conditions (all *t* < 1.4, *p* > .1), indicating that their abnormal performance on the learning task was not due to difficulty in discriminating visually between the exemplars.

## 4 Discussion

The ATLs are thought to play a central role in the representation of conceptual knowledge (Lambon Ralph et al., 2010, Patterson et al., 2007). Here, we investigated how damage to the ATLs affects acquisition of new concepts. SD patients completed a category learning task, in which the category members conformed to a family resemblance structure designed to replicate the key computational challenges of acquiring real-world concepts. The patients were able to learn some information about the stimuli but did so in a sub-optimal fashion that differed from healthy controls in systematic and theoretically important ways. For optimal performance, it was necessary to integrate all three critical dimensions of the stimuli into a coherent representation. Patients were unable to do this and instead based all of their category judgements on a single dimension. This deficit is consistent with the hub-and-spoke theory of conceptual knowledge and specifically with the theory that the ATLs act as a pan-modal representational hub, which integrates a concept's disparate sensory-motor and verbal features into a single coherent representation (Lambon Ralph et al., 2010, Rogers et al., 2004). With damage to the ATLs, SD patients largely retained the ability to associate *individual* stimulus features with novel categories but were unable to acquire the integrated feature structure necessary for optimal discrimination between the two categories.

SD patients also demonstrated over-generalisation of the successful learning in their preferred dimension: information from one dimension dominated category decisions, even when the other features of the stimulus pointed towards an alternative response. This over-generalisation of remaining knowledge is also common when SD patients attempt to make use of their remaining conceptual knowledge in everyday life and in clinical assessment (Lambon Ralph and Patterson, 2008, Lambon Ralph et al., 2010). Over the course of the disease, patients become increasingly likely to over-extend category boundaries on the basis of superficial characteristics (e.g., accepting a butterfly as a type of bird; Mayberry et al., 2011), to use a single, highly familiar concept label to refer to a whole class of items (e.g., all forms of fruit may be called “apples”; Hodges, Graham, & Patterson, 1995), and to imbue items with over-generalised, stereotypical attributes in delayed-copy drawing (e.g., the case of the four-legged duck; Bozeat et al., 2003, Lambon Ralph and Howard, 2000). In the present study, we were able to unmask one of the basic mechanisms underpinning this profound deterioration in conceptual representation: cerebral atrophy in SD affects integrated conceptual representations that bind together the various sources of information that characterise a particular set of items. Without these coherent concepts, classification and identification of objects comes to depend on superficial surface characteristics.

Interestingly, another study indicates that SD patients can successfully make category judgements about novel items when they are not required to form integrated representations. Koenig et al. (2006) investigated six SD patients' ability to classify novel stimuli based on a category membership rule and on similarity to a prototype. Koenig et al.'s study differs from ours in that Koenig et al. explicitly provided patients with the appropriate rule to apply or prototype to compare during categorisation. In contrast, we required patients to learn the relevant category structure themselves through feedback. Patients in the Koenig et al. study performed similarly to controls and the authors attributed this good performance to intact attentional and executive processes. One possibility for the difference between the two studies is that the application of explicit rules to determine category membership depends heavily on executive and attentional processes, while the acquisition of multi-dimensional feature structure is a more automatic process involving implicit learning mechanisms in temporal regions. This assertion is supported by an investigation in healthy participants, on which the present learning task was based (Waldron & Ashby, 2001). As in our study, participants were trained to classify stimuli without being given any explicit instruction regarding the structure of the category. They were trained with category structures in which a single feature determined category membership as well ones that required integration of features. Crucially, an executively-demanding concurrent task slowed learning of the single-feature categories but had little effect on the categories that required integration. The authors suggested that learning a single-feature category involved using executive resources to extract an explicit rule that governs category membership. In contrast, learning of the feature-integration categories was assumed to be an implicit stimulus-driven process (see also Ashby & Ell, 2001). Relating these findings to our patient group, it appears that while integration of features was impaired, executively-mediated rule extraction was intact in most cases, hence their over-learning of a single feature dimension. However, the two most severe patients (N.H. and E.T.) were less successful in acquiring appropriate single-feature information, perhaps indicating a decline in executive processes as the disease progresses.

Which regions within the ATLs are critically involved in acquiring and storing coherent concepts? In SD, atrophy affects the entire ATL region, though it is concentrated in polar and ventrolateral regions (Gorno-Tempini et al., 2004, Mion et al., 2010). Converging evidence from other methodologies has also implicated the ventral and lateral aspects of the ATLs in the representation of conceptual knowledge (Binney et al., 2010, Marinkovic et al., 2003, Pobric et al., 2007, Visser and Lambon Ralph, 2011). A parallel line of work has implicated medial anterior temporal regions, particularly the perirhinal cortex, in the perception and learning of novel feature conjunctions, both in humans (Barense et al., 2005, Taylor et al., 2006) and non-human primates (Bussey et al., 2002, Murray and Richmond, 2001). Damage to this region is associated with deficits in discriminating between novel stimuli based on conjunctions of their features. Medial and ventrolateral temporal regions also appear to interact in the acquisition and representation of concepts. For example, neurons in both the perirhinal and ventrolateral ATLs change their response characteristics as monkeys learn novel visual associations, suggesting that both areas are involved (Messinger, Squire, Zola, & Albright, 2001). It is likely that medial temporal regions play a critical role in the perception and initial encoding of new conceptual information, while ventrolateral temporal cortex is necessary for longer-term storage of concepts (Albright, 2012, Squire et al., 2004). Established theories of learning hold that this division of labour is necessary to avoid catastrophic interference between similar representations (McClelland, McNaughton, & O'Reilly, 1995). It is also consistent with the data observed in this study. SD patients were not generally amnesic for novel information, as would be expected in patients with medial temporal lobe dysfunction: they were able to learn single-feature information and maintain this between the two training sessions. Nor were they impaired in perceptual discriminations based on conjunctions of features (though another study has shown that SD patients can be impaired on such discriminations for meaningful items; Barense et al., 2010). In contrast, their deficits stemmed from an inability to extract the underlying patterns of feature co-occurrence present over many trials to form representations of the two stimulus categories. However, a great deal more work is needed to determine precisely how different sub-regions within the ATLs work together to process complex feature conjunctions in a single experience and to integrate information acquired over many experiences into coherent concepts. The striatum and putamen are also involved in learning to classify stimuli when integration of two dimensions is required, particularly in the early stages of learning (Waldschmidt & Ashby, 2011). These subcortical structures are intact in SD (Mummery et al., 2000) but their interaction with the damaged temporal cortex has not been investigated.

In this study, we focused on the integration of stimulus features within the visual modality. However, it is important to note that the ATLs play an important role in integrating conceptual knowledge *across* modalities: they are equally activated during conceptual processing of visual and auditory stimuli, both verbally and non-verbally (Binney et al., 2010, Spitsyna et al., 2006, Visser and Lambon Ralph, 2011). In the primate literature, the ATLs have been associated with associative learning both within the visual modality (Albright, 2012, Messinger et al., 2001) and across different sensory modalities (Murray and Richmond, 2001, Parker and Gaffan, 1998). Indeed, the ATLs are strongly connected to visual, auditory and other sensory cortices (Moran et al., 1987, Pandya and Seltzer, 1982), making this region a key area of polysensory or “transmodal” cortex (Mesulam, 1998, Patterson et al., 2007, Simmons and Barsalou, 2003). The hub-and-spoke model distinguishes between this transmodal cortex and spoke regions that are sensitive to structure in a single modality, though this distinction may be relative rather than absolute. Recently, we have proposed that the anterior temporal region acts as a graded representational space (Plaut, 2002), in which the type of information coded by each area of cortex is determined by the inputs it receives from sensory and unimodal association cortices (Binney et al., 2012). For example, the dorsolateral ATL receives strong input from the posterior superior temporal gyrus, leading this area to exhibit relative specialisation for information in auditory and verbal modalities (Visser & Lambon Ralph, 2011). Ventromedial ATL is strongly connected with ventral occipitotemporal cortex, leading to a prominent role in coding visual properties. Critically, between these extremes lies equi-modal cortex in the inferior temporal and fusiform gyri that responds similarly across modalities and presumably codes transmodal structure. In summary, the process of extracting meaning from our experience with objects involves the fusion of complex sets of information from sensory inputs, motor programmes and verbal experience. We have demonstrated that one key aspect of this process, the integration of individual features into coherent concepts, depends critically on the ATLs.

## Footnotes

1 With the exception of P.W., there appears to be a tendency towards classification based on number rather than the other two dimensions. It is not clear whether this occurred by chance or if reflects a consistent tendency for SD patients to focus on quantity above other perceptual features. There was no such bias towards number in the control group.
